# Internet addiction among young Bangladeshi adults: critical commentary on Hassan et al. (2020)

**DOI:** 10.1186/s42506-020-00054-x

**Published:** 2020-09-29

**Authors:** Mark D. Griffiths, Mohammed A. Mamun

**Affiliations:** 1 Psychology Department, Nottingham Trent University, 50 Shakespeare Street, Nottingham, NG1 4FQ UK; 2 Undergraduate Research Organization, Savar, Dhaka, Bangladesh

Dear Editor,

We were very interested to read the paper by Hassan et al. [1] examining the prevalence and associated factors of internet addiction (IA) among young Bangladeshi adults. However, there are a number of issues of concern that we would like to raise.

In the first paragraph, Hassan et al. claimed that *“Kimberly Young was the first to introduce the concept of internet addiction disorder (IAD) in 1996”* (p. 1). The paper that was cited [2] was published in December 1996. However, it was actually Griffiths who published the first paper on internet addiction in November 1996 [3] having already published a more general paper on “technological addictions” in February 1995 [4] as well as populist article on internet addiction in April 1995 [5].

They then cited a paper by Xin et al. [6] to provide worldwide prevalence figures (who simply reported prevalence data from other studies) but did not make any reference to the largest meta-analysis in the IA field by Cheng and Li [7] who examined prevalence figures comprising 164 studies (*N* = 89,281) from 31 nations in seven world regions, and producing a global estimate of 6% for IA. Hassan et al. mentioned the wide variations in IA prevalence across different world regions but provided no explanation as to why. Such variation in global IA rates can be due to a number of factors, the most important of which are arguably methodological (e.g., there are many different psychometric instruments used to assess IA, and different researchers use different cutoff points even when the same instrument is being used) [8]. These important factors were not mentioned.

One of the major omissions in the paper is that in their Introduction, not a single previous Bangladeshi study was discussed even though there are many (e.g., [8–21] - see Table 1 for details), and only one was mentioned in the Discussion [10]. Hasan also claimed that: *“Most of the studies conducted previously evaluated the prevalence of internet addiction and its predictors in adolescent samples, within the age range of 12 to 18 years”* (p. 2). There was no reference for this assertion, and furthermore, it is simply not true. For instance, a systematic review by Kuss et al. [22] of 68 large-scale studies of IA (i.e., studies with over 1000 participants) reported that 24 of the studies had purely adult samples, and a further 15 studies had age ranges that included participants over the age of 18 years. In sum, this systematic review showed that in the large-scale studies in the period they examined, 39 of the 68 studies were not carried out on 12–18-year age group samples.

**Table 1:** Comparison of Bangladeshi studies examining internet addiction prevalence (in alphabetical order)

Authors (year published)	Study location	Study population	Sample size (age range)	Assessment tool	Scale response	Total scale items	Cutoff scores used	Main findings
**Afrin et al. (2017)** [19]	Chittagong	High school students	279 (14–17 years)	Internet Addiction Survey	Yes/no	9	< 3 = normal internet user; 4 to 6 = moderate internet user; ≤ 7 = severe user	2.5% severely addicted to the internet, 64.9% moderately addicted to the internet
**Hassan et al. (2020)** [1]	Chittagong, Dhaka, Sylhet	Adults	454 (19–35 years)	Internet Addiction Test	5-point Likert-type scale ranging from 1 (rarely) to 5 (always)	20*	20–49 points = average internet user; ≥ 50 points = internet addicted	27.1% prevalence of internet addiction
**Islam and Hossin (2016)** [10]	Dhaka	University students	573 (20–30 years)	Internet Addiction Test	5-point Likert-type scale ranging from 1 (rarely) to 5 (always)	20	≥ 50 = moderate, excessive, or problematic internet user	24% problematic internet users
**Jahan et al. (2019)** [20]	Dhaka	University medical students	390 (18–26 years)	Internet Addiction Survey	Yes/no	9	< 3 = normal internet user; 4 to 6 = moderate internet user; ≤ 7 = severe user	31.5% normal users, 49.2% moderately addicted users, and 19.3% severely internet addicted users
**Karim and Nigar (2014)** [15]	Dhaka	University students	177 (18–25 years)	Bangla Internet Addiction Test	5-point Likert-type scale ranging from 1 (rarely) to 5 (always)	18	< 36 = minimal internet user; 36–62 = moderate internet user; > 62 = excessive internet user	63.95% minimal internet users, 34.3% moderate internet users, 1.7% excessive internet users
**Khan (2012)** [18]	Dhaka	High school students	797 (mean age = 16.5 years)	Internet Addiction Test	5-point Likert-type scale ranging from 1 (rarely) to 5 (always)	20	Not reported	20.20% reported as having “internet addiction disorder”
**Mamun and Griffiths (2019)** [11]	Dhaka	University students	300 (mean age = 20.7 years)	Bergen Facebook Addiction Scale	5-point Likert-type scale ranging from 1 (very rarely) to 5 (very often)	6	≥ 18 = at risk of being addicted to Facebook	39.7% at risk of being addicted to Facebook
**Mamun et al. (2019)** [16]	Dhaka	University students	405 (mean age = 20.2 years)	Internet Addiction Test	5-point Likert-type scale ranging from 1 (rarely) to 5 (always)	20	≥ 50 = moderate to high or problematic internet user	32.6% problematic internet users
**Mamun et al. (2019)** [14]	Rajshahi	Graduated students	284 (mean age = 21.1 years)	Internet Addiction Test	5-point Likert-type scale ranging from 1 (rarely) to 5 (always)	20	< 60 = non-excessive internet users; ≥ 60 = excessive internet users	0% internet addicts, but 3.9% classed as excessive users
**Mostafa et al. (2019)** [21]	Chittagong	Medical and university students	379 (18–30 years)	Internet Addiction Test	5-point Likert-type scale ranging from 1 (rarely) to 5 (always)	20	< 20 = normal internet user; 20–49 = mild internet user; 50–79 = moderate internet user; 80–100 = severe internet user	Majority of the participants were mild problematic users (54.88%). The prevalence of internet addiction was 1.06% (severe users)
**Siddiqi et al. (2018)** [12]	Dhaka	High school students	376 (13–19 years)	Bergen Facebook Addiction Scale	5-point Likert-type scale ranging from 1 (rarely) to 5 (always)	6**	≥ 20 = at risk of being addicted to Facebook	49.8%, 41.0%, and 1.6% mild, moderate, and severe Facebook addicted and 7.6% non-problematic users
**Uddin et al. (2016)** [13]	Dhaka	University students	475 (18–25 years)	Internet Addiction Test	5-point Likert-type scale ranging from 1 (rarely) to 5 (always)	20	≤ 30 = normal internet user; 31–49 = mild internet user; 50–79 = moderate internet user; ≥ 80 = severe or excessive internet user	47.7% male and 44.5% female students severely addicted to the internet, 7.1% male and 33.9% female students moderately addicted to the internet, 20.7% male and 7.7% female students mildly addicted to the internet

*Paper said the Bangla Internet Addiction Test was used, but based on the description, it appears the Internet Addiction Test was used

**Paper claimed there were 18 items, but the Bergen Facebook Addiction Scale only has six items

Another major criticism of Hassan et al.’s study is their scoring on the scale used for IA assessment. The authors said they used the 20-item Bangla version of the Internet Addiction Test (IAT) with a cutoff score of 50 (out of 100) to categorize the participants as internet addicts. However, the Bangla IAT is an 18-item scale (whereas the original English language IAT has 20 items) with total scores ranging from 20 to 90 [15]. The scoring for Bangla IAT is 18–35 for minimal use, 36–62 for moderate use, and 63–90 for excessive use. However, Hassan et al. did not use the appropriate scoring for the validated Bangla IAT, and given they used a 20-item scale, it is not even clear if they used the Bangla IAT at all (because there is no 20-item validated version). Additionally, they cited two papers [15, 23] who they claimed had used a cutoff score of 50 to class individuals as internet addicts but neither of these two psychometric studies suggested 50 as a cutoff score.

Hassan et al. also wrote that the IAT *“has been scientifically analyzed to state an ambiguous psychometric factor structure”* (p. 2). On reading this, we were unsure whether the authors deliberately meant to say the factor structure was “ambiguous” (as opposed to “unambiguous”) given that they were putting forward the rationale for using the IAT in the first place. However, we would agree that there is no consensus on the psychometric properties of the IAT, because previous studies have reported markedly different factor structures [23–28]. Furthermore, the items for the IAT were developed in 1998 and a number of the items are now very out of date given the rise of smartphones and social media. These are reasons that would weaken the rationale for using the IAT rather than strengthen it.

Hassan et al.’s study reported that over a quarter of their Bangladeshi sample are addicted to the internet (27.1%). Given a cutoff score of 50 was used to classify individuals as being “addicted” to the internet, it is not surprising the percentage of internet addiction was so high because the vast majority will not have been addicted with such a low cutoff score. There is simply no face validity to the claim that over one quarter of Bangladeshi adults are addicted to the internet. The original developer of the IAT initially suggested a score of above 80 to be classed as an internet addict [29]. Hassan et al. then compared their Bangladeshi prevalence rate to a single previous Bangladeshi study. To our knowledge, at the time of writing, there were at least 14 previous papers specifically examining problematic internet use in Bangladesh (i.e., 10 papers on internet addiction, two on social media addiction, and two commentaries), and of these, 11 papers examined online addiction prevalence in Bangladesh, yet apart from one study [10], none of these was referred to. Hassan et al. then compared their IA prevalence rates to just four other seemingly arbitrary studies in four different countries (i.e., Jordan, Iran, UK, and Taiwan) out of the hundreds that have now been published. The lack of comparison with studies that are clearly relevant (particularly previous Bangladeshi studies) is of concern. Put simply, the “Discussion” did not contextualize the findings in relation to the most relevant studies.

Additionally, Hassan et al. claimed in their conclusion that *“[t]he prevalence of excessive internet use is significant among young adults in Bangladesh, which is conforming with the global trend”* (p. 7). However, few studies were cited in the paper on which to base such a comparison. Furthermore, *“excessive internet use”* is not the same as internet addiction given that many excessive internet users are not addicted to the internet (14).

Hassan et al. also provided what they claimed to be study strengths (i.e., sample from various Bangladeshi regions, utilizing a *“high number of sociodemographic variables as well as variables related to internet use behavior and regular activity”* [p. 6]) without referring to any previous Bangladeshi papers as a benchmark. Confusingly, the authors used a survey to collect the data, but then in the 'Strengths and limitations' section, they said the participants were *“conveniently selected for the interview”* (p. 6).

Based on these aforementioned criticisms, it can be concluded that the paper by Hassan et al. [1] has many methodological and conceptual weaknesses as well as including a number of assertions that were just simply and factually incorrect.

## Data Availability

Not applicable

## Abbreviations

IA: Internet addiction
IAD: Internet addiction disorder
IAT: Internet Addiction Test
